# 1-(4-Bromo­phen­yl)ferrocene

**DOI:** 10.1107/S1600536808031759

**Published:** 2008-10-09

**Authors:** Vincent O. Nyamori, Muhammad D. Bala

**Affiliations:** a School of Chemistry, University of KwaZulu-Natal, Westville Campus, Private Bag X54001, Durban 4000, South Africa

## Abstract

In the title compound, [Fe(C_5_H_5_)(C_11_H_8_Br)], the distance of the Fe atom from the centroids of the unsubstituted and substituted cyclo­penta­dienyl (Cp) rings is 1.644 (1) and 1.643 (1) Å, respectively. The ferrocenyl moiety deviates from an eclipsed geometry, with marginally tilted Cp rings and an inter­planar angle between the Cp and benzene rings of 13.0 (4)°. The crystal structure is stabilized by C—H⋯π inter­actions between a cyclo­penta­dienyl H atom and the cyclo­penta­dienyl ring of a neighbouring mol­ecule.

## Related literature

For related literature, see: Allen (2002 ▶); Anderson *et al.* (2003 ▶); Cambridge Crystallographic Data Centre (2002 ▶); Coe *et al.* (1994 ▶); Hor *et al.* (1991 ▶); Imrie *et al.* (2002 ▶, 2003 ▶); Knoesen & Lotz (1999 ▶); Togni & Hayashi (1995 ▶).
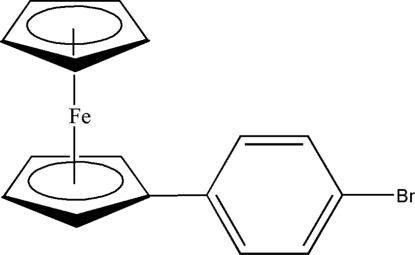


## Experimental

### 

#### Crystal data

[Fe(C_5_H_5_)(C_11_H_8_Br)]
*M* *_r_* = 341.02
Monoclinic, 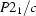

*a* = 16.4991 (3) Å
*b* = 9.9578 (2) Å
*c* = 7.9269 (1) Å
β = 97.084 (1)°
*V* = 1292.41 (4) Å^3^
*Z* = 4
Mo *K*α radiation
μ = 4.24 mm^−1^
*T* = 173 (2) K
0.37 × 0.32 × 0.07 mm

#### Data collection

Bruker APEXII CCD area-detector diffractometer
Absorption correction: integration (*XPREP*; Bruker, 2005 ▶) *T*_min_ = 0.303, *T*_max_ = 0.756
15480 measured reflections
3126 independent reflections
2775 reflections with *I* > 2σ(*I*)
*R*_int_ = 0.039

#### Refinement

*R*[*F* ^2^ > 2σ(*F* ^2^)] = 0.030
*wR*(*F* ^2^) = 0.075
*S* = 1.15
3126 reflections
163 parameters
H-atom parameters constrained
Δρ_max_ = 0.53 e Å^−3^
Δρ_min_ = −0.40 e Å^−3^

### 

Data collection: *APEX2* (Bruker, 2005 ▶); cell refinement: *SAINT-Plus* (Bruker, 2005 ▶); data reduction: *SAINT-Plus*; program(s) used to solve structure: *SHELXTL* (Sheldrick, 2008 ▶); program(s) used to refine structure: *SHELXTL*; molecular graphics: *PLATON* (Spek, 2003 ▶) and *ORTEP-3* (Farrugia, 1997 ▶); software used to prepare material for publication: *SHELXTL*.

## Supplementary Material

Crystal structure: contains datablocks global, I. DOI: 10.1107/S1600536808031759/lx2069sup1.cif

Structure factors: contains datablocks I. DOI: 10.1107/S1600536808031759/lx2069Isup2.hkl

Additional supplementary materials:  crystallographic information; 3D view; checkCIF report

## Figures and Tables

**Table 1:** Hydrogen-bond geometry (Å, °) *Cg*1 is the centroid of the C1–C5 cyclopentadienyl ring.

*D*—H⋯*A*	*D*—H	H⋯*A*	*D*⋯*A*	*D*—H⋯*A*
C2—H2⋯*Cg*1^i^	0.95	2.90	3.780 (4)	154

Symmetry code: (i) 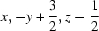.

**Fig. 1.**
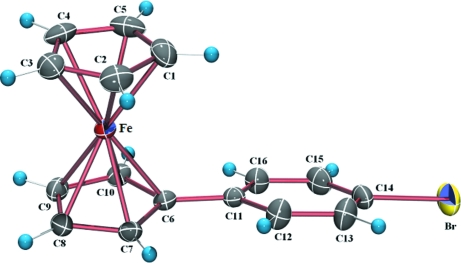
Molecular structure of the title complex with the atom labelling scheme. Ellipsoids are drawn at the 50% probability level.

**Fig. 2.**
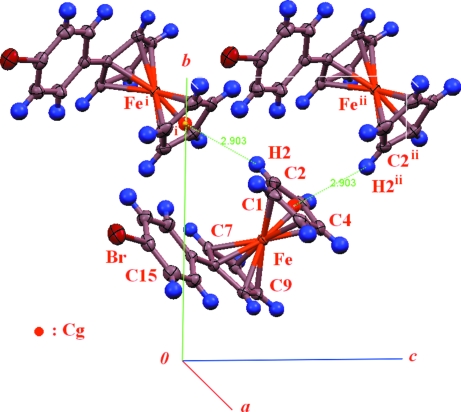
A stereoview of the interactions in the crystal structure of (I). [Symmetry code: (i) x, -y+3/2, z-1/2; (ii) x, -y+3/2, z+1/2.]

## supplementary crystallographic information

### Comment

Ferrocene compounds especially those synthesized by reacting a *para*-substituted phenylferrocene 4-Fc—C_6_H*_4_*-*R* (*R* = any atom or group) are of great interest in the field of material chemistry (Togni & Hayashi, 1995). They are employed as precursors in the synthesis of ferrocenomesogens with the ferrocenyl moiety incorporated as a terminal group (Imrie *et al.*, 2002, 2003). Interest in these compounds stems from their potential use either as metathesis catalysts or therapeutic radiopharmaceuticals (Hor *et al.*, 1991; Knoesen & Lotz, 1999). The compounds have also been used to synthesize non-linear optical materials containing molybdenum or tungsten redox centres (Coe *et al.*,1994).

In the title compound (I, Fig. 1), the distance of the Fe atom from the centroids of the unsubstituted (C1—C5) and the substituted (C6—C10) cyclopentadienyl rings are 1.644 (1) and 1.643 (1) Å respectively, indicating that the *para-* substitution (bromophenyl group) has little influence on Fe—Cp bonding interactions. The two Cp rings deviate from an eclipsed conformation with torsion angles around 11.0 (2)°. The rings are also marginally tilted towards each other with a tilt angle between the planes of the two rings of 0.83 (2)°. The interplanar angle between the Cp and the phenyl rings of (I) was 13.0 (4)°. This value is very close to the 12.8° observed by Anderson *et al.* (2003) as the median value upon analysis of 17 structures from the April 2002 version of the Cambridge Structural Database using *ConQuest* Version 1.4 (Allen, 2002). The molecular packing (Fig. 2) is stabilized by C—H···π interactions between a cyclopentadienyl H atom and the cyclopentadienyl ring of an adjacent molecule, with a C2—H2···Cg^i^ separation of 2.90 Å (Fig. 2 and Table 1; Cg is the centroid of the C1-C5 cyclopentadienyl ring, symmetry code as in Fig. 2).

### Experimental

The title compound (I) was synthesized *via* the diazonium reaction as follows: A solution of 4-bromobenzene diazonium sulfate was prepared by the reaction of 4-bromoaniline (20.02 g, 0.12 mol) in dilute sulfuric acid (100 cm^3^) to which sodium nitrite (11.65 g, 0.17 mol) was slowly added in water at 278 K. The reaction temperature was continually monitored and held at 278 K during the addition. The resultant solution was filtered and the filtrate was immediately added to a cold, well stirred solution of ferrocene(24.60 g, 0.13 mol) in diethyl ether (450 cm^3^). Stirring was continued at 278 K for 3 h and then at room temperature for a further 12 h. The ether layer was separated, washed with water, dried over anhydrous sodium sulfate and evaporated. The residue was purified by column chromatography on silica gel. Hexane was used to elute unreacted ferrocene and the product was eluted from the column using 1 : 1 hexane : dichloromethane mixture to yield 4.57 g, 11% of pure (I). mp 122–123 °C; Spectroscopic analysis: IR *ν*_max_(KBr/cm^-1^) 3086, 3053, 2925, 2853, 1588, 1509, 1446, 1406, 1383, 1278, 1103, 1088, 1066,1050, 1030, 1001, 884, 819; ^1^H NMR (CDCl_3_, 300 MHz) δ_H_ 7.41 (2*H*, d, *J* 8.5, ArH), 7.34 (2*H*, d, *J *8.5, ArH), 4.62 (2*H*, t, *J* 1.8, C_5_H_4_),4.34 (2*H*, t, *J* 1.8, C_5_H_4_), 4.04 (5*H*, s, C_5_H_5_);

EI–MS 70 eV *m/z *343 (18), 342 (96), 341 (22), 340 (*M*^+^, 100), 260 (3), 205 (23), 203 (10), 202 (9); Elemental analysis (Found: C, 56.4; H, 3.8%; *M*, 339.9551. required for C_16_H_13_FeBr: C, 56.6; H, 3.9%; *M*, 339.9550).

### Refinement

All H atoms attached to C atoms were fixed geometrically and treated as riding with C—H = 0.95 Å and *U*_iso_(H) = 1.2*U*_eq_(C).

### Crystal data

**Table:**

[Fe(C_5_H_5_)(C_11_H_8_Br)]	*F*(000) = 680
*M**_r_* = 341.02	*D*_x_ = 1.753 Mg m^−^^3^
Monoclinic, *P*2_1_/*c*	Melting point = 395–396 K
Hall symbol: -P 2ybc	Mo *K*α radiation, λ = 0.71073 Å
*a* = 16.4991 (3) Å	Cell parameters from 8414 reflections
*b* = 9.9578 (2) Å	θ = 2.4–28.4°
*c* = 7.9269 (1) Å	µ = 4.24 mm^−^^1^
β = 97.084 (1)°	*T* = 173 K
*V* = 1292.41 (4) Å^3^	Plate, orange
*Z* = 4	0.37 × 0.32 × 0.07 mm

### Data collection

**Table:**

Bruker APEXII CCD area-detector diffractometer	3126 independent reflections
Radiation source: fine-focus sealed tube	2775 reflections with *I* > 2σ(*I*)
graphite	*R*_int_ = 0.039
Detector resolution: 10.0 pixels mm^-1^	θ_max_ = 28.0°, θ_min_ = 1.2°
φ and ω scans	*h* = −21→21
Absorption correction: integration (XPREP; Bruker, 2005)	*k* = −13→13
*T*_min_ = 0.303, *T*_max_ = 0.756	*l* = −10→10
15480 measured reflections	

### Refinement

**Table:**

Refinement on *F*^2^	Primary atom site location: structure-invariant direct methods
Least-squares matrix: full	Secondary atom site location: difference Fourier map
*R*[*F*^2^ > 2σ(*F*^2^)] = 0.030	Hydrogen site location: inferred from neighbouring sites
*wR*(*F*^2^) = 0.075	H-atom parameters constrained
*S* = 1.15	*w* = 1/[σ^2^(*F*_o_^2^) + (0.0124*P*)^2^ + 2.8125*P*] where *P* = (*F*_o_^2^ + 2*F*_c_^2^)/3
3126 reflections	(Δ/σ)_max_ = 0.001
163 parameters	Δρ_max_ = 0.53 e Å^−^^3^
0 restraints	Δρ_min_ = −0.40 e Å^−^^3^

### Special details

**Table:**

Geometry. All esds (except the esd in the dihedral angle between two l.s. planes) are estimated using the full covariance matrix. The cell esds are taken into account individually in the estimation of esds in distances, angles and torsion angles; correlations between esds in cell parameters are only used when they are defined by crystal symmetry. An approximate (isotropic) treatment of cell esds is used for estimating esds involving l.s. planes.
Refinement. Refinement of F^2^ against ALL reflections. The weighted R-factor wR and goodness of fit S are based on F^2^, conventional R-factors R are based on F, with F set to zero for negative F^2^. The threshold expression of F^2^ > 2sigma(F^2^) is used only for calculating R-factors(gt) etc. and is not relevant to the choice of reflections for refinement. R-factors based on F^2^ are statistically about twice as large as those based on F, and R- factors based on ALL data will be even larger.

### Fractional atomic coordinates and isotropic or equivalent isotropic displacement parameters (Å^2^)

**Table:**

	*x*	*y*	*z*	*U*_iso_*/*U*_eq_	
Br	−0.055964 (19)	0.43903 (4)	−0.29425 (5)	0.04324 (11)	
Fe	0.35189 (2)	0.48664 (4)	0.27601 (5)	0.01850 (9)	
C1	0.27574 (19)	0.6354 (3)	0.3370 (4)	0.0346 (7)	
H1	0.2256	0.6612	0.2732	0.042*	
C2	0.3538 (2)	0.6885 (3)	0.3185 (4)	0.0307 (7)	
H2	0.3653	0.7560	0.2401	0.037*	
C3	0.41137 (19)	0.6237 (3)	0.4365 (4)	0.0308 (6)	
H3	0.4686	0.6397	0.4520	0.037*	
C4	0.3689 (2)	0.5298 (3)	0.5287 (4)	0.0334 (7)	
H4	0.3925	0.4720	0.6167	0.040*	
C5	0.2854 (2)	0.5381 (3)	0.4659 (4)	0.0360 (7)	
H5	0.2428	0.4865	0.5043	0.043*	
C6	0.29438 (16)	0.3883 (3)	0.0676 (3)	0.0208 (5)	
C7	0.36479 (16)	0.4587 (3)	0.0264 (3)	0.0225 (5)	
H7	0.3652	0.5272	−0.0568	0.027*	
C8	0.43450 (17)	0.4078 (3)	0.1324 (4)	0.0261 (6)	
H8	0.4894	0.4363	0.1313	0.031*	
C9	0.40733 (18)	0.3072 (3)	0.2393 (4)	0.0258 (6)	
H9	0.4408	0.2569	0.3226	0.031*	
C10	0.32169 (18)	0.2951 (3)	0.2001 (4)	0.0237 (6)	
H10	0.2879	0.2350	0.2529	0.028*	
C11	0.20996 (16)	0.4044 (3)	−0.0135 (3)	0.0213 (5)	
C12	0.18754 (19)	0.5119 (3)	−0.1218 (4)	0.0309 (6)	
H12	0.2269	0.5785	−0.1392	0.037*	
C13	0.1081 (2)	0.5237 (3)	−0.2055 (4)	0.0340 (7)	
H13	0.0933	0.5970	−0.2797	0.041*	
C14	0.05166 (17)	0.4264 (3)	−0.1775 (4)	0.0295 (6)	
C15	0.07133 (19)	0.3200 (3)	−0.0704 (4)	0.0337 (7)	
H15	0.0314	0.2545	−0.0526	0.040*	
C16	0.15020 (18)	0.3097 (3)	0.0114 (4)	0.0285 (6)	
H16	0.1640	0.2364	0.0862	0.034*	

### Atomic displacement parameters (Å^2^)

**Table:**

	*U*^11^	*U*^22^	*U*^33^	*U*^12^	*U*^13^	*U*^23^
Br	0.02264 (15)	0.0582 (2)	0.0466 (2)	0.00362 (14)	−0.00493 (13)	−0.00758 (17)
Fe	0.02091 (18)	0.01753 (18)	0.01723 (19)	−0.00139 (14)	0.00303 (14)	−0.00126 (14)
C1	0.0301 (15)	0.0309 (16)	0.0423 (19)	0.0082 (13)	0.0019 (13)	−0.0127 (14)
C2	0.0456 (18)	0.0172 (13)	0.0304 (16)	−0.0021 (12)	0.0082 (14)	−0.0022 (11)
C3	0.0297 (15)	0.0293 (15)	0.0330 (16)	−0.0040 (12)	0.0022 (12)	−0.0130 (12)
C4	0.054 (2)	0.0295 (15)	0.0171 (14)	0.0031 (14)	0.0049 (13)	−0.0048 (11)
C5	0.0425 (18)	0.0341 (17)	0.0358 (18)	−0.0090 (14)	0.0226 (14)	−0.0120 (14)
C6	0.0250 (13)	0.0199 (12)	0.0178 (13)	−0.0013 (10)	0.0036 (10)	−0.0017 (10)
C7	0.0237 (13)	0.0271 (14)	0.0174 (13)	−0.0009 (11)	0.0057 (10)	−0.0008 (10)
C8	0.0214 (13)	0.0325 (15)	0.0243 (14)	0.0014 (11)	0.0026 (11)	−0.0061 (11)
C9	0.0294 (14)	0.0239 (13)	0.0231 (14)	0.0055 (11)	−0.0015 (11)	−0.0036 (11)
C10	0.0313 (14)	0.0180 (12)	0.0217 (14)	−0.0025 (11)	0.0023 (11)	−0.0018 (10)
C11	0.0231 (13)	0.0225 (13)	0.0187 (13)	−0.0003 (10)	0.0037 (10)	−0.0032 (10)
C12	0.0292 (15)	0.0301 (15)	0.0325 (16)	−0.0060 (12)	−0.0003 (12)	0.0061 (12)
C13	0.0328 (16)	0.0331 (16)	0.0337 (17)	0.0012 (13)	−0.0051 (13)	0.0047 (13)
C14	0.0180 (12)	0.0402 (17)	0.0296 (16)	0.0027 (12)	0.0007 (11)	−0.0085 (13)
C15	0.0250 (14)	0.0360 (16)	0.0406 (18)	−0.0068 (13)	0.0061 (13)	−0.0038 (14)
C16	0.0275 (14)	0.0272 (14)	0.0307 (16)	−0.0028 (11)	0.0038 (12)	0.0026 (12)

### Geometric parameters (Å, °)

**Table:**

Br—C14	1.902 (3)	C6—C7	1.429 (4)
Fe—C3	2.033 (3)	C6—C10	1.432 (4)
Fe—C4	2.034 (3)	C6—C11	1.469 (4)
Fe—C5	2.035 (3)	C7—C8	1.431 (4)
Fe—C7	2.035 (3)	C7—H7	0.9500
Fe—C2	2.037 (3)	C8—C9	1.420 (4)
Fe—C8	2.038 (3)	C8—H8	0.9500
Fe—C1	2.039 (3)	C9—C10	1.414 (4)
Fe—C10	2.043 (3)	C9—H9	0.9500
Fe—C9	2.044 (3)	C10—H10	0.9500
Fe—C6	2.050 (3)	C11—C12	1.394 (4)
C1—C5	1.403 (5)	C11—C16	1.396 (4)
C1—C2	1.417 (4)	C12—C13	1.399 (4)
C1—H1	0.9500	C12—H12	0.9500
C2—C3	1.405 (4)	C13—C14	1.381 (4)
C2—H2	0.9500	C13—H13	0.9500
C3—C4	1.424 (5)	C14—C15	1.371 (5)
C3—H3	0.9500	C15—C16	1.384 (4)
C4—C5	1.408 (5)	C15—H15	0.9500
C4—H4	0.9500	C16—H16	0.9500
C5—H5	0.9500		
			
C3—Fe—C4	40.98 (13)	Fe—C3—H3	126.1
C3—Fe—C5	68.36 (13)	C5—C4—C3	107.6 (3)
C4—Fe—C5	40.50 (14)	C5—C4—Fe	69.79 (18)
C3—Fe—C7	126.85 (12)	C3—C4—Fe	69.49 (17)
C4—Fe—C7	165.51 (13)	C5—C4—H4	126.2
C5—Fe—C7	152.17 (14)	C3—C4—H4	126.2
C3—Fe—C2	40.37 (13)	Fe—C4—H4	126.1
C4—Fe—C2	68.37 (13)	C1—C5—C4	108.4 (3)
C5—Fe—C2	68.20 (13)	C1—C5—Fe	69.99 (17)
C7—Fe—C2	107.11 (12)	C4—C5—Fe	69.71 (17)
C3—Fe—C8	107.68 (12)	C1—C5—H5	125.8
C4—Fe—C8	127.68 (13)	C4—C5—H5	125.8
C5—Fe—C8	165.74 (14)	Fe—C5—H5	126.1
C7—Fe—C8	41.13 (11)	C7—C6—C10	107.1 (2)
C2—Fe—C8	118.32 (13)	C7—C6—C11	126.9 (2)
C3—Fe—C1	68.17 (13)	C10—C6—C11	126.0 (2)
C4—Fe—C1	68.10 (14)	C7—C6—Fe	68.97 (15)
C5—Fe—C1	40.30 (14)	C10—C6—Fe	69.28 (15)
C7—Fe—C1	118.13 (13)	C11—C6—Fe	128.33 (19)
C2—Fe—C1	40.69 (13)	C6—C7—C8	108.0 (2)
C8—Fe—C1	152.45 (14)	C6—C7—Fe	70.09 (15)
C3—Fe—C10	153.13 (12)	C8—C7—Fe	69.56 (16)
C4—Fe—C10	119.09 (12)	C6—C7—H7	126.0
C5—Fe—C10	108.57 (12)	C8—C7—H7	126.0
C7—Fe—C10	68.70 (11)	Fe—C7—H7	125.9
C2—Fe—C10	165.43 (12)	C9—C8—C7	108.1 (2)
C8—Fe—C10	68.36 (12)	C9—C8—Fe	69.89 (16)
C1—Fe—C10	127.81 (13)	C7—C8—Fe	69.32 (15)
C3—Fe—C9	119.06 (12)	C9—C8—H8	126.0
C4—Fe—C9	108.37 (12)	C7—C8—H8	126.0
C5—Fe—C9	128.08 (13)	Fe—C8—H8	126.4
C7—Fe—C9	68.88 (11)	C10—C9—C8	108.0 (2)
C2—Fe—C9	152.56 (13)	C10—C9—Fe	69.72 (16)
C8—Fe—C9	40.70 (12)	C8—C9—Fe	69.41 (16)
C1—Fe—C9	165.56 (13)	C10—C9—H9	126.0
C10—Fe—C9	40.48 (11)	C8—C9—H9	126.0
C3—Fe—C6	164.67 (12)	Fe—C9—H9	126.5
C4—Fe—C6	152.67 (12)	C9—C10—C6	108.7 (2)
C5—Fe—C6	118.56 (12)	C9—C10—Fe	69.81 (16)
C7—Fe—C6	40.94 (10)	C6—C10—Fe	69.77 (15)
C2—Fe—C6	126.92 (12)	C9—C10—H10	125.6
C8—Fe—C6	68.94 (11)	C6—C10—H10	125.6
C1—Fe—C6	107.50 (12)	Fe—C10—H10	126.4
C10—Fe—C6	40.95 (11)	C12—C11—C16	117.9 (3)
C9—Fe—C6	68.80 (11)	C12—C11—C6	121.3 (2)
C5—C1—C2	108.1 (3)	C16—C11—C6	120.8 (3)
C5—C1—Fe	69.70 (18)	C11—C12—C13	121.2 (3)
C2—C1—Fe	69.60 (17)	C11—C12—H12	119.4
C5—C1—H1	126.0	C13—C12—H12	119.4
C2—C1—H1	126.0	C14—C13—C12	118.4 (3)
Fe—C1—H1	126.3	C14—C13—H13	120.8
C3—C2—C1	107.9 (3)	C12—C13—H13	120.8
C3—C2—Fe	69.66 (17)	C15—C14—C13	121.9 (3)
C1—C2—Fe	69.71 (17)	C15—C14—Br	119.2 (2)
C3—C2—H2	126.0	C13—C14—Br	118.9 (2)
C1—C2—H2	126.0	C14—C15—C16	119.0 (3)
Fe—C2—H2	126.2	C14—C15—H15	120.5
C2—C3—C4	108.0 (3)	C16—C15—H15	120.5
C2—C3—Fe	69.96 (17)	C15—C16—C11	121.5 (3)
C4—C3—Fe	69.53 (17)	C15—C16—H16	119.2
C2—C3—H3	126.0	C11—C16—H16	119.2
C4—C3—H3	126.0		
			
C3—Fe—C1—C5	−81.8 (2)	C9—Fe—C6—C10	37.07 (17)
C4—Fe—C1—C5	−37.5 (2)	C3—Fe—C6—C11	−79.1 (5)
C7—Fe—C1—C5	156.93 (19)	C4—Fe—C6—C11	68.0 (4)
C2—Fe—C1—C5	−119.4 (3)	C5—Fe—C6—C11	34.3 (3)
C8—Fe—C1—C5	−168.4 (2)	C7—Fe—C6—C11	−121.0 (3)
C10—Fe—C1—C5	73.0 (2)	C2—Fe—C6—C11	−48.8 (3)
C9—Fe—C1—C5	41.0 (6)	C8—Fe—C6—C11	−159.0 (3)
C6—Fe—C1—C5	113.8 (2)	C1—Fe—C6—C11	−8.0 (3)
C3—Fe—C1—C2	37.54 (19)	C10—Fe—C6—C11	120.1 (3)
C4—Fe—C1—C2	81.9 (2)	C9—Fe—C6—C11	157.2 (3)
C5—Fe—C1—C2	119.4 (3)	C10—C6—C7—C8	0.4 (3)
C7—Fe—C1—C2	−83.7 (2)	C11—C6—C7—C8	−177.7 (2)
C8—Fe—C1—C2	−49.0 (3)	Fe—C6—C7—C8	59.43 (19)
C10—Fe—C1—C2	−167.57 (17)	C10—C6—C7—Fe	−59.00 (18)
C9—Fe—C1—C2	160.4 (4)	C11—C6—C7—Fe	122.8 (3)
C6—Fe—C1—C2	−126.84 (18)	C3—Fe—C7—C6	−167.24 (17)
C5—C1—C2—C3	−0.1 (3)	C4—Fe—C7—C6	163.3 (4)
Fe—C1—C2—C3	−59.4 (2)	C5—Fe—C7—C6	−51.6 (3)
C5—C1—C2—Fe	59.3 (2)	C2—Fe—C7—C6	−127.18 (17)
C4—Fe—C2—C3	38.05 (19)	C8—Fe—C7—C6	119.1 (2)
C5—Fe—C2—C3	81.8 (2)	C1—Fe—C7—C6	−84.50 (19)
C7—Fe—C2—C3	−127.35 (18)	C10—Fe—C7—C6	38.03 (16)
C8—Fe—C2—C3	−84.2 (2)	C9—Fe—C7—C6	81.59 (17)
C1—Fe—C2—C3	119.2 (3)	C3—Fe—C7—C8	73.7 (2)
C10—Fe—C2—C3	161.7 (4)	C4—Fe—C7—C8	44.2 (5)
C9—Fe—C2—C3	−50.4 (3)	C5—Fe—C7—C8	−170.7 (2)
C6—Fe—C2—C3	−168.12 (17)	C2—Fe—C7—C8	113.71 (18)
C3—Fe—C2—C1	−119.2 (3)	C1—Fe—C7—C8	156.39 (18)
C4—Fe—C2—C1	−81.1 (2)	C10—Fe—C7—C8	−81.08 (18)
C5—Fe—C2—C1	−37.4 (2)	C9—Fe—C7—C8	−37.51 (17)
C7—Fe—C2—C1	113.5 (2)	C6—Fe—C7—C8	−119.1 (2)
C8—Fe—C2—C1	156.65 (19)	C6—C7—C8—C9	−0.4 (3)
C10—Fe—C2—C1	42.5 (5)	Fe—C7—C8—C9	59.35 (19)
C9—Fe—C2—C1	−169.5 (2)	C6—C7—C8—Fe	−59.76 (18)
C6—Fe—C2—C1	72.7 (2)	C3—Fe—C8—C9	114.28 (18)
C1—C2—C3—C4	0.0 (3)	C4—Fe—C8—C9	73.3 (2)
Fe—C2—C3—C4	−59.4 (2)	C5—Fe—C8—C9	42.8 (6)
C1—C2—C3—Fe	59.4 (2)	C7—Fe—C8—C9	−119.4 (2)
C4—Fe—C3—C2	−119.1 (3)	C2—Fe—C8—C9	156.84 (17)
C5—Fe—C3—C2	−81.4 (2)	C1—Fe—C8—C9	−169.2 (2)
C7—Fe—C3—C2	71.7 (2)	C10—Fe—C8—C9	−37.46 (16)
C8—Fe—C3—C2	113.20 (19)	C6—Fe—C8—C9	−81.58 (18)
C1—Fe—C3—C2	−37.82 (19)	C3—Fe—C8—C7	−126.30 (17)
C10—Fe—C3—C2	−169.9 (2)	C4—Fe—C8—C7	−167.27 (17)
C9—Fe—C3—C2	156.04 (18)	C5—Fe—C8—C7	162.2 (5)
C6—Fe—C3—C2	38.5 (5)	C2—Fe—C8—C7	−83.74 (19)
C5—Fe—C3—C4	37.7 (2)	C1—Fe—C8—C7	−49.8 (3)
C7—Fe—C3—C4	−169.18 (18)	C10—Fe—C8—C7	81.96 (17)
C2—Fe—C3—C4	119.1 (3)	C9—Fe—C8—C7	119.4 (2)
C8—Fe—C3—C4	−127.70 (19)	C6—Fe—C8—C7	37.84 (16)
C1—Fe—C3—C4	81.3 (2)	C7—C8—C9—C10	0.2 (3)
C10—Fe—C3—C4	−50.8 (3)	Fe—C8—C9—C10	59.22 (19)
C9—Fe—C3—C4	−84.9 (2)	C7—C8—C9—Fe	−59.00 (19)
C6—Fe—C3—C4	157.6 (4)	C3—Fe—C9—C10	157.07 (17)
C2—C3—C4—C5	0.0 (3)	C4—Fe—C9—C10	113.59 (18)
Fe—C3—C4—C5	−59.6 (2)	C5—Fe—C9—C10	72.8 (2)
C2—C3—C4—Fe	59.6 (2)	C7—Fe—C9—C10	−81.54 (17)
C3—Fe—C4—C5	118.8 (3)	C2—Fe—C9—C10	−168.1 (2)
C7—Fe—C4—C5	155.7 (4)	C8—Fe—C9—C10	−119.4 (2)
C2—Fe—C4—C5	81.3 (2)	C1—Fe—C9—C10	40.2 (6)
C8—Fe—C4—C5	−168.89 (19)	C6—Fe—C9—C10	−37.49 (16)
C1—Fe—C4—C5	37.35 (19)	C3—Fe—C9—C8	−83.5 (2)
C10—Fe—C4—C5	−84.8 (2)	C4—Fe—C9—C8	−126.99 (18)
C9—Fe—C4—C5	−127.73 (19)	C5—Fe—C9—C8	−167.72 (18)
C6—Fe—C4—C5	−48.5 (3)	C7—Fe—C9—C8	37.89 (16)
C5—Fe—C4—C3	−118.8 (3)	C2—Fe—C9—C8	−48.7 (3)
C7—Fe—C4—C3	36.9 (6)	C1—Fe—C9—C8	159.7 (5)
C2—Fe—C4—C3	−37.50 (18)	C10—Fe—C9—C8	119.4 (2)
C8—Fe—C4—C3	72.3 (2)	C6—Fe—C9—C8	81.94 (17)
C1—Fe—C4—C3	−81.5 (2)	C8—C9—C10—C6	0.0 (3)
C10—Fe—C4—C3	156.36 (18)	Fe—C9—C10—C6	59.08 (19)
C9—Fe—C4—C3	113.45 (19)	C8—C9—C10—Fe	−59.03 (19)
C6—Fe—C4—C3	−167.3 (2)	C7—C6—C10—C9	−0.3 (3)
C2—C1—C5—C4	0.1 (3)	C11—C6—C10—C9	177.9 (2)
Fe—C1—C5—C4	59.3 (2)	Fe—C6—C10—C9	−59.10 (19)
C2—C1—C5—Fe	−59.2 (2)	C7—C6—C10—Fe	58.80 (18)
C3—C4—C5—C1	−0.1 (3)	C11—C6—C10—Fe	−123.0 (3)
Fe—C4—C5—C1	−59.5 (2)	C3—Fe—C10—C9	−48.9 (3)
C3—C4—C5—Fe	59.4 (2)	C4—Fe—C10—C9	−84.5 (2)
C3—Fe—C5—C1	81.3 (2)	C5—Fe—C10—C9	−127.49 (19)
C4—Fe—C5—C1	119.5 (3)	C7—Fe—C10—C9	82.01 (18)
C7—Fe—C5—C1	−47.8 (3)	C2—Fe—C10—C9	157.9 (4)
C2—Fe—C5—C1	37.72 (19)	C8—Fe—C10—C9	37.67 (17)
C8—Fe—C5—C1	157.7 (5)	C1—Fe—C10—C9	−168.24 (18)
C10—Fe—C5—C1	−127.14 (19)	C6—Fe—C10—C9	120.0 (2)
C9—Fe—C5—C1	−168.01 (18)	C3—Fe—C10—C6	−168.9 (2)
C6—Fe—C5—C1	−83.5 (2)	C4—Fe—C10—C6	155.50 (17)
C3—Fe—C5—C4	−38.18 (19)	C5—Fe—C10—C6	112.47 (18)
C7—Fe—C5—C4	−167.3 (2)	C7—Fe—C10—C6	−38.02 (16)
C2—Fe—C5—C4	−81.8 (2)	C2—Fe—C10—C6	37.8 (5)
C8—Fe—C5—C4	38.2 (6)	C8—Fe—C10—C6	−82.37 (17)
C1—Fe—C5—C4	−119.5 (3)	C1—Fe—C10—C6	71.7 (2)
C10—Fe—C5—C4	113.35 (19)	C9—Fe—C10—C6	−120.0 (2)
C9—Fe—C5—C4	72.5 (2)	C7—C6—C11—C12	−12.5 (4)
C6—Fe—C5—C4	156.95 (18)	C10—C6—C11—C12	169.7 (3)
C3—Fe—C6—C7	42.0 (5)	Fe—C6—C11—C12	78.7 (3)
C4—Fe—C6—C7	−171.0 (2)	C7—C6—C11—C16	165.4 (3)
C5—Fe—C6—C7	155.38 (18)	C10—C6—C11—C16	−12.4 (4)
C2—Fe—C6—C7	72.3 (2)	Fe—C6—C11—C16	−103.4 (3)
C8—Fe—C6—C7	−38.01 (16)	C16—C11—C12—C13	−1.1 (5)
C1—Fe—C6—C7	113.01 (18)	C6—C11—C12—C13	176.9 (3)
C10—Fe—C6—C7	−118.9 (2)	C11—C12—C13—C14	0.4 (5)
C9—Fe—C6—C7	−81.79 (17)	C12—C13—C14—C15	0.3 (5)
C3—Fe—C6—C10	160.8 (4)	C12—C13—C14—Br	−178.6 (2)
C4—Fe—C6—C10	−52.1 (3)	C13—C14—C15—C16	−0.3 (5)
C5—Fe—C6—C10	−85.8 (2)	Br—C14—C15—C16	178.6 (2)
C7—Fe—C6—C10	118.9 (2)	C14—C15—C16—C11	−0.4 (5)
C2—Fe—C6—C10	−168.88 (17)	C12—C11—C16—C15	1.0 (4)
C8—Fe—C6—C10	80.85 (18)	C6—C11—C16—C15	−176.9 (3)
C1—Fe—C6—C10	−128.13 (18)		

### Hydrogen-bond geometry (Å, °)

**Table:**

*D*—H···*A*	*D*—H	H···*A*	*D*···*A*	*D*—H···*A*
C2—H2···Cg1^i^	0.95	2.90	3.780 (4)	154

Symmetry codes: (i) *x*, −*y*+3/2, *z*−1/2.
